# Harnessing regulatory T cell neuroprotective activities for treatment of neurodegenerative disorders

**DOI:** 10.1186/s13024-020-00375-7

**Published:** 2020-06-05

**Authors:** Jatin Machhi, Bhavesh D. Kevadiya, Ijaz Khan Muhammad, Jonathan Herskovitz, Katherine E. Olson, R. Lee Mosley, Howard E. Gendelman

**Affiliations:** 1 Department of Pharmacology and Experimental Neuroscience, Center for Neurodegenerative Disorders, University of Nebraska Medical Center, Omaha, NE 68198-5880 USA; 2 Department of Radiology, School of Medicine, Stanford University, Palo Alto, 94304 USA; 3 Department of Pharmacy, University of Swabi, Anbar Swabi, 23561 Pakistan; 4 Department of Pathology and Microbiology, University of Nebraska Medical Center, Omaha, NE 68198-5880 USA

**Keywords:** Regulatory T cells (Tregs), Effector T cells (Teffs), Dendritic cells, Microglia, Immune transformation, Neurodegenerative disorders

## Abstract

**Abstract:**

Emerging evidence demonstrates that adaptive immunity influences the pathobiology of neurodegenerative disorders. Misfolded aggregated self-proteins can break immune tolerance leading to the induction of autoreactive effector T cells (Teffs) with associated decreases in anti-inflammatory neuroprotective regulatory T cells (Tregs). An imbalance between Teffs and Tregs leads to microglial activation, inflammation and neuronal injury. The cascade of such a disordered immunity includes the drainage of the aggregated protein antigens into cervical lymph nodes serving to amplify effector immune responses. Both preclinical and clinical studies demonstrate transformation of this altered immunity for therapeutic gain. We posit that the signs and symptoms of common neurodegenerative disorders such as Alzheimer’s and Parkinson’s diseases, amyotrophic lateral sclerosis, and stroke can be attenuated by boosting Treg activities.

**Graphical Abstract:**

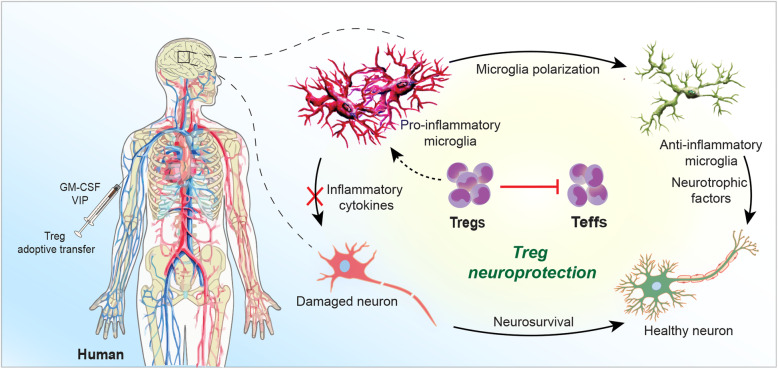

## Highlights

- Misfolded self-proteins can break immune tolerance.
- Dominant effector T cell responses can affect disease onset and progression.
- Effector T cells can exacerbate microglial inflammation.
- Regulatory T cells suppress adaptive and innate effectors.
- Boosting regulatory T cell frequency and function serve as a neuroprotective role.

## Background

As of now, only symptomatic therapies are available to abate the signs and symptoms of neurodegenerative disorders. This lack of disease-combating neurorestorative agents has promoted research efforts into finding the means to attenuate disease. One of these therapies builds on the correction of peripheral-brain immune asymmetry through harnessing the powers of adaptive immunity [1–3]. For decades disease mitigating immune responses were studied for their abilities to find then eliminate infectious and cancerous threats. However, what was not known, until the recent decade, was that they can also affect the pathobiology of central nervous system (CNS) disease. The loss of CNS immune privilege has forged new investigations into the ways neurodegenerative disorders can be treated [3–5]. Indeed, it is now accepted that adaptive immunity can affect brain development and homeostasis. For example, deficiencies in CD4+ T cell number and function lead to impaired neurogenesis and cognition [6, 7]. Also, restoring a balance between divergent T cell subsets can sustain neuronal homeostasis and preclude disease-inciting pro-inflammatory events [2, 8]. Progressive neurodegenerative, neuroinflammatory, and neuroinfectious disease rests, in measure, around adaptive immunity. All are sped by elevated effector and downregulated regulatory T cells (Teffs and Tregs) [5, 9]. However, past attempts to correct such aberrant host immunity using immunosuppressive agents have failed to affect disease outcomes [10, 11]. This led to initial discouragements in immune-based approaches for CNS disease by some neuroimmunologists. Indeed, by some due diligence, a new therapeutic frontier has emerged designed to transform neurotoxic Teffs into neuroprotective Tregs. This led to the idea that the microenvironment of the diseased brain could be altered through the control of disease-inciting neuroinflammation. A new created microenvironment was made that served to slow or even prevent disease progression [2, 12]. The idea was restoring disease altered CNS immune homeostasis. This was accomplished by breaking the chain link between systemic neuroinflammation and neural injury [4, 5] through immune transformation.

All together, CNS disorders are intertwined with basic immune processes of antigen presentation, cytokine secretion, leukocyte recruitment, cell activation, macromolecular lymphatic drainage, and inflammation. How immunity and CNS disease are linked remained a black box in trying to explain the notion of both detrimental and restorative immunity. It was found that the control of specific T cell functional phenotypes affect disease control [3–5, 13, 14]. While adaptive immunity has broad biologic and physiological consequences to the nervous system [2, 9, 15] how it can be tamed in developing therapeutic strategies proved key in establishing a bridge between T cell function and brain repair. This proved complex as it is based on the brain’s ever changing microenvironment and the evolution of immune functions [10, 11].

What is now broadly accepted is that activation of peripheral immunity, regardless of cause, triggers neural tissue repair and controls the tempo of CNS injury [1, 2, 5, 16]. Both innate and adaptive immunity can incite neuroinflammation and accelerate aggregated misfolded protein accumulation that lead to neuronal demise. Such pathogenic events are operative, at varying degrees, in Alzheimer’s and Parkinson’s diseases (AD and PD), amyotrophic lateral sclerosis (ALS), multiple sclerosis (MS), and stroke [1, 3, 12, 17–21]. In contrast, peripheral immunity also facilitates brain repair [2, 3, 9], in part, by suppression of neurotoxic Teff responses [1, 22–26]. Therapeutic immune transformers include, but are not limited to, glatiramer acetate, anti-CD3, rapamycin, granulocyte-macrophage colony stimulating factor (GM-CSF), histone deacetylase inhibitors (HDACi), vasoactive intestinal peptide (VIP), VIP receptor-2 agonist, interleukin-2 (IL-2), and snake and bee venoms [1, 22, 24–30]. All induce substantive neuroprotective Tregs. To these ends, we review how Tregs serve to sustain brain homeostasis in variant neurodegenerative conditions.

## CNS immunity and T cells

Peripheral immunocytes [T cells, B cells, natural killer (NK) cells, and dendritic cells (DCs)] are present in the brain parenchyma, the meninges, and the choroid plexus. There they can serve as either neuroprotectants or disease instigators depending upon the local environment [4, 5]. CD4+ to CD8+ cells patrol the cerebrospinal fluid (CSF) in search of cognate antigen and upon recognition induce local memory effector immune responses [31, 32]. Also, antigen-presenting cells (APCs) macrophages, and DCs distributed in meningeal, choroid plexus, and perivascular compartments, work in tandem, to affect T cell neuroimmunity [4, 33]. While searching for cognate antigen, T cell interactions with innate microglia and astrocytes also affect CNS homeostasis. In the absence of antigen recognition and activation, T cells return to the periphery through the CNS lymphatic draining system into the deep cervical lymph nodes. In the healthy brain, entry and exit of circulating immunocytes are greatly controlled to maintain homeostatic neuronal function. However, during inflammation (Fig. 2), immunocytes and CNS antigens interactions in secondary lymph nodes affect the blood-brain-barrier (BBB, composed of astrocytic end feet and parenchymal basal lamina) cell trafficking and secretory activities [4]. Leakiness in the BBB and blood-CSF barriers leads to altered transporter and cytokine responses that allow excessive transmigration of immune cells into the brain with secondary pro-inflammatory activities [5, 9, 11, 34]. While antigen-specific CD4+ T cells cross the BBB [35], it is their antigen recognition that induces immune responses known to affect the upregulation of trafficking molecules, cell transmigration, and neural integrity [4]. When T cells do not recognize cognate antigen, they do not cross the BBB and undergo apoptosis [36]. T cells constantly patrol for cognate antigen in the CNS draining lymph nodes. Peripheral T cell effects on brain pathology are notable as removal of their draining lymph nodes significantly affects disease severity [37]. Even without crossing the BBB, T cells maintain brain function through a cascade of immune signaling and secreted molecules [38–40]. Therefore, the coordinated immune responses, in and outside the CNS, have broad functional consequences to the brain.

The notion that immune cells and macromolecules have limited access inside the CNS is no longer believed [41, 42]. Recent studies re-identified prior works demonstrating a structural and functional glymphatic system serving to control waste management [14, 43, 44]. The passing of CSF from the subarachnoid space through the arterial paravascular space to the brain interstitium collects cellular waste through the aquaporin water channel. The resulting CSF flow moves toward the venous perivascular space and brings waste into the meningeal lymphatic vessels where it drains into secondary lymphoid tissue [43–46]. This glymphatic system facilitates brain clearance of both hydrophilic and lipophilic compounds and is pivotal in the removal of neurotoxic protein aggregates like amyloid-beta (Aβ). When the glymphatic system becomes dysfunctional, it plays a key role in AD pathobiology [47]. Cellular macromolecules also leave the CNS by CSF drainage from the cribriform plate into nasal lymphatic vasculature. These enter the subarachnoid space to reach the meningeal lymphatics that deliver materials to deep cervical lymph nodes [4]. Both cellular waste-draining CSF and immune cells drain through the meningeal lymphatic system into peripheral deep cervical lymph nodes to activate and prime T cells [5, 33, 48].

**Fig. 1:**
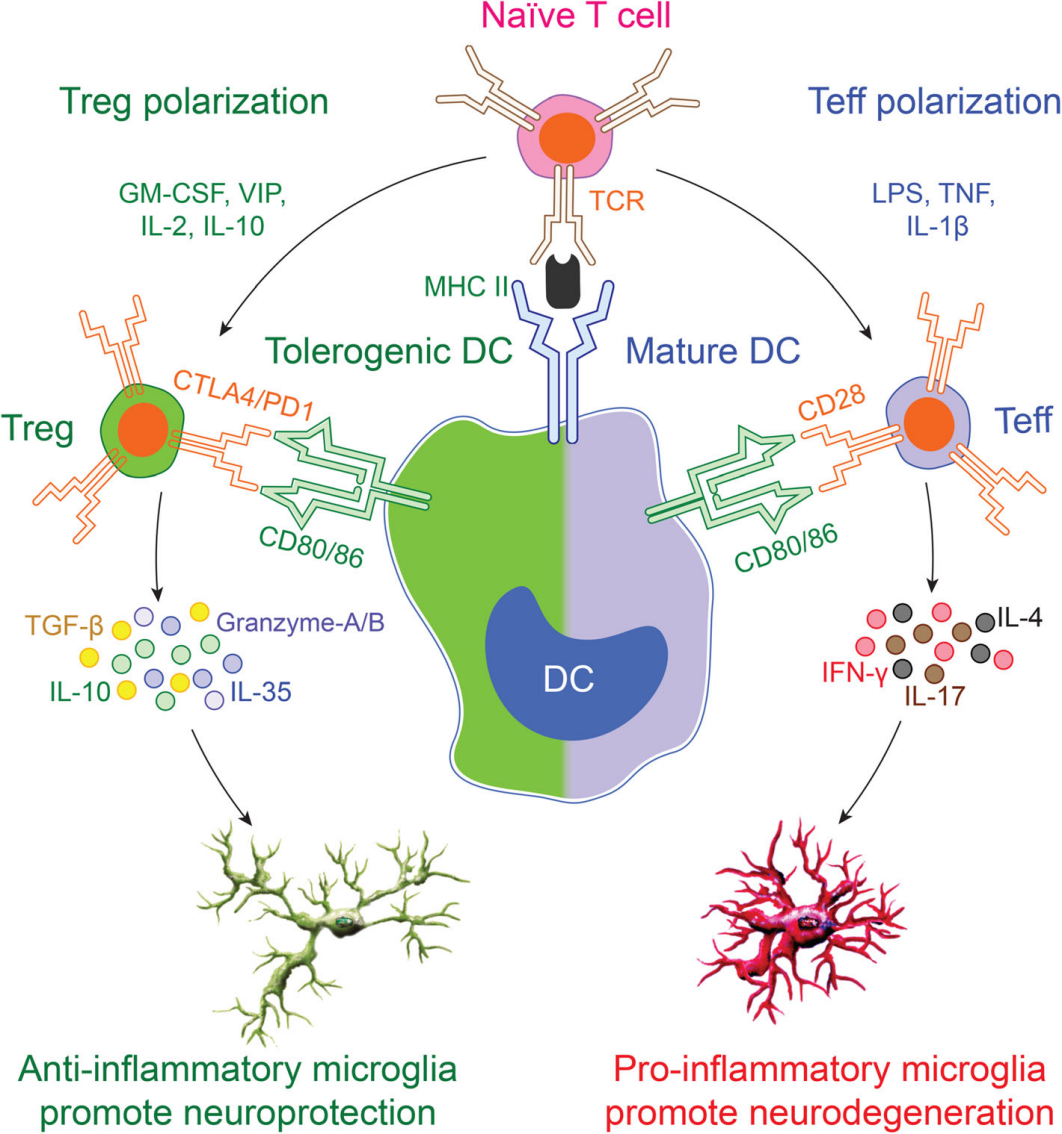
DCs and T cell polarization. Immature DCs take up antigen, process and present it to immunocytes. Antigen uptake induces maturation signals in DCs in cooperation with the upregulation of co-stimulatory molecules. Mature DCs encounter naïve T cells through MHCII-T cell receptor (TCR) interactions, leading to T cell activation, Teff differentiation, and secretion of pro-inflammatory molecules (IL-10, IL-35, TGF-β, granzymes, etc.). In contrast, some DCs can maintain central and peripheral immune tolerance called tolerogenic DCs. The tolerogenic DCs exhibit low levels of costimulatory molecules and as such, provide insufficient stimulatory signals to naïve T cells to induce Treg and anti-inflammatory cytokines (IL-4, IL17, IFN-γ, etc.). Tregs suppress Teff function and proliferation to maintain immune tolerance. Teffs secreted molecules govern pro-inflammatory microglia polarization. In contrast, Tregs favor anti-inflammatory microglia polarization, supporting neuroprotection. Balance between Teffs and Tregs is essential to maintain homeostasis while their imbalance leads to neurodegeneration through microglia responses

T cells develop in the thymus through the expression of T cell receptors and glycoproteins. CD8 and CD4 glycoprotein expressing cells are naïve CD8+ and CD4+ T cells. Upon activation, the latter, CD4+ T cells, differentiate into effector subsets exerting diverse immune responses. These subsets include T helper 1 (Th1), Th2, Th9, Th17, Th22, follicular helper T cells (Tfh), and Tregs. Each CD4+ subset secretes cytokines with either pro- or anti-inflammatory functions. Th1 cells secrete IL-2, interferon gamma (IFN-γ), and tumor necrosis factor (TNF); Th2 cells secrete IL-4, IL-5, IL-10 and IL-13; Th9 cells secrete IL-9; Th17 cells secrete IL-17, and Tregs secrete IL-10 and transforming growth factor beta (TGF-β). Different cytokines secreted from CD4+ subsets drive effector or regulatory immune responses. Among different CD4+ effector subsets, Th1 and Th17 cells drive pro-inflammatory responses while Tregs exert anti-inflammatory and immunosuppressive activities. A balance between both pro- and anti-inflammatory immune responses is essential to maintain generalized homeostasis and especially within the CNS where an imbalance can lead to disease [2, 49]. Except Tregs, although all the CD4+ T cells are considered effector subtypes, herein, pro-inflammatory and neurodestructive functions are included in groups of Th1 and Th17 cells defined as Teffs, whereas Treg functions are described as anti-inflammatory, immunosuppressive and neuroprotective (Fig. 1).

## Microglia

**Fig. 2:**
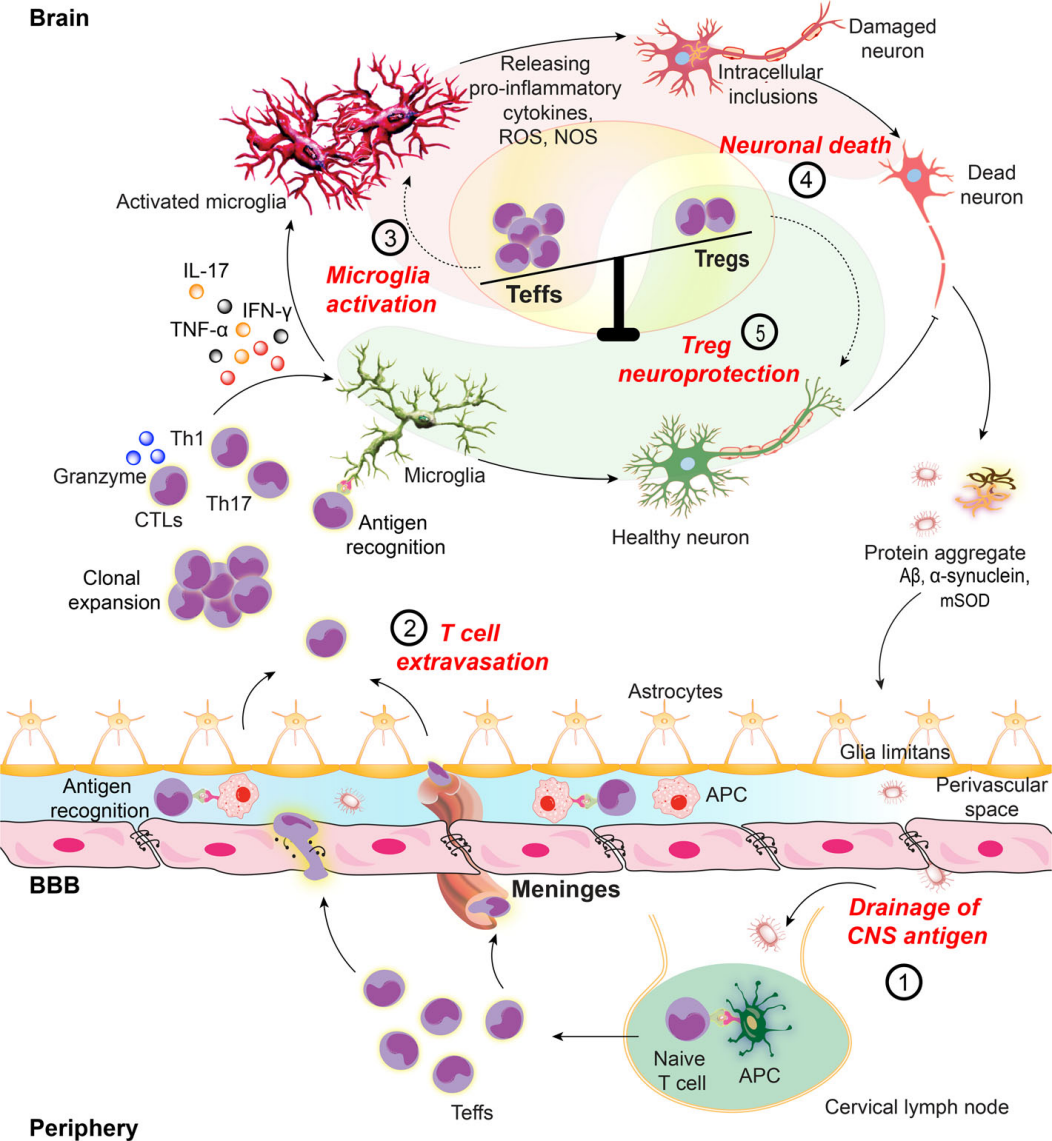
Teff activities promote neuroinflammation. In neurodegenerative disorders, CNS antigens including Aβ, α-syn, mSOD and MBP provoke microglia immune responses leading to neuroinflammatory cascade in affected brain regions. (1) Self-antigen or misfolded proteins are generated from damaged neuronal cells. The neural antigens drain to the peripheral lymphoid nodes by meningeal lymphatic vessels where they are taken up by local APCs including macrophages and DCs. Natural self-antigens are presented to peripheral T cells in MHCII dependent manner. (2) Naïve T cells, upon recognition of cognate antigen, differentiate into antigen-specific Teffs. Reactive microglia secrete cytokine-chemokine milieu to upregulate cell adhesion molecules (CAM) by blood-brain barrier (BBB) endothelial cells, opening the gate for peripheral primed T cells. Teffs (Th1 and Th17) with upregulated integrins and CAM ligands readily cross the BBB. Teffs also cross the blood-CSF barrier through choroid plexus meninges. After extravasation into the brain, Teffs are reactivated upon recognition of cognate antigen on common CNS APCs. These include perivascular macrophages (PVMs), choroid plexus and meningeal macrophages and DCs and parenchymal microglia. (3, 4) Activated Teffs secrete pro-inflammatory and neurotoxic mediators to polarize microglia to a higher activation state, producing pro-inflammatory cytokines and reactive oxygen and nitrogen species which further perpetuate the inflammatory cascade to induce neurotoxicity. (5) Tregs maintain immune tolerance by suppressing effector immune responses. Naïve T cells can also differentiate into Tregs upon recognition of cognate antigen on peripheral APCs in secondary lymphoid tissues. Differentiated Tregs exert neuroprotective responses through multiple mechanisms. The inflammatory immune responses observed in neurodegenerative disorders are the outcome of Teff-Treg imbalance with upregulated Teff responses

Microglia are CNS innate immune cells which play indispensable roles in affecting neuronal homeostasis and in promoting T cells responses in the brain. Macrophages, the prototype peripheral innate immune cell adopt M1 (classically pro-inflammatory activated) and M2 (alternatively anti-inflammatory activated) phenotypes [50]. Similarly, microglia also exhibit functional plasticity that leads to their altered cell number, morphology, surface receptor expression, and growth factor and cytokine secretion [51]. Microglia shift into an M1 or an M2 phenotype depending on the surrounding immune environment [52]. Pro-inflammatory microglia secrete increased TNF-α, IL-6, IL-1β, neurotoxic reactive oxygen species (ROS), nitric oxide, and reduced neurotrophic factors that additively injure neuronal cells [53, 54] (Fig. 2). Whereas, anti-inflammatory microglia secrete IL-4, IL-13, TGF-β, and neurotrophic factors, like insulin-like growth factor 1 (IGF-1) that induce phagocytosis of protein aggregates collectively attributing to neuroprotection [53, 54]. Astrocytes, another abundant innate immune cell, also play diverse anatomical and functional roles inside the CNS [55]. Like microglia, astrocytes in neuroinflammatory and ischemic conditions acquire A1 and A2 phenotypes that secret neurotoxic pro-inflammatory mediators and neurotrophic factors, respectively. However, these secreted molecules are not well-identified [56–58].

Microglia phenotypes are not absolute and the cell’s morphology and functional changes that occur during physiological and diverse pathological conditions determine outcomes. Studies of human postmortem microglia revealed that due to the brain’s microenvironment, they acquire multiple phenotypic signatures affected by location [59]. Markers for microglial proliferation and activation, for example, are seen readily in the subventricular zone and thalamus. These reflect regional heterogeneity. Immune profiling of postmortem human microglia are generally comparable to cells freshly isolated and from other species [60]. However, during disease, such as experimental allergic encephalomyelitis (EAE), microglial subsets are linked to brain function and disease. Two subsets are critical for brain homeostasis while four others are disease associated. All microglial subsets share the P2RY12^lo^TMEM119^lo^MD-1^hi^ profile and express specific chemokine, cytokine and cysteine receptors and proteases that suggest broad cellular dynamics [61]. Heterogeneous microglia are seen in Huntington’s disease, EAE and ALS demonstrating unique molecular signaling and cytokine signatures [62]. Although the advanced high throughput single-cell RNAseq and mass cytometry have uncovered microglial heterogeneity in the setting of disease and homeostatic function, whether it is sufficient to define different subtypes is unknown. The identification of unique microglial function associated with each subset at disease may define the role microglia play in health and disease [63, 64]. Likewise, astrocyte phenotypes are not absolute; and it is still unclear what possible signaling pathways are involved [56, 57]. Both, microglia and astrocytes under normal circumstances recognize pathogenic signals and respond through different cytokine and chemokine milieu that ultimately determines immune responses [65].

## Gut microbiota and neuroimmunity

The dynamic population of microorganisms, including bacteria, viruses, fungi, and archaea, that reside inside the gastrointestinal tract play an indispensable role in homeostasis. Alterations in the composition of the gut microbiota called dysbiosis, lead to the development of metabolic, autoimmune, and neurodegenerative diseases [66, 67]. Recent studies established the link between gut dysbiosis and inflammatory changes of different organs including the brain [66, 68].

Human and animal model studies have shown that increased frequency of pathological bacteria or absence of useful bacterial components in the gut associated with neurodegenerative diseases including MS, AD, PD and ALS. A study with 34 pairs of discordant MS twins found differences in the gut microbiota composition compared to healthy counterparts. Furthermore, MS twin-derived microbiota increased disease severity in animal models that develop spontaneous brain autoimmunity, compared to the healthy twin-derived microbiota [69].

Gut dysbiosis is also implicated in AD pathogenesis. Recently, fecal microbiota compositions of AD patients and age- and sex-matched normal controls were found significantly different. Several bacterial taxa including *Bacteroides*, *Actinobacteria*, *Ruminococcus*, *Lachnospiraceae*, and *Selenomonadales* were different in AD patients from controls [70]. In AD animal studies, intestinal dysbiosis speeds cognitive decline and neurodegeneration [71, 72]. In PD patients, gut dysbiosis with elevated harmful microbial taxa including *Proteus mirabilis* promoted dopaminergic neuronal death and motor impairment [73]. PD patients derived microbiota enhanced severity of motor symptoms in α-synuclein-overexpressing mice compared to healthy donor microbiota. The short-chain fatty acids (SCFA) produced from gut microbiota may activate certain immune cells that promote α-synuclein aggregation and microgliosis to impair motor symptoms [68]. On the other hand, butyrate producing bacteria, *Butyrivibrio fibrisolvens* were found selectively reduced in the gut microbiota of mice genetically susceptible to ALS. Here butyrate treatment significantly attenuated disease severity [74]. Neuroprotective effects of butyrate were also reported in the 1-methyl-4-phenyl-1,2,3,6-tetrahydropyridine (MPTP) model of PD [75, 76].

CD4+ T cells mediate crosstalk between gut microbiota and the CNS. Microbiota and their secreted molecules including SCFA, neurotransmitters, and other metabolites affect differentiation and expansion of pro- and anti-inflammatory CD4+ T cells. Commensal microbes, such as segmented filamentous bacterium, induce pro-inflammatory Th17 cells [77] while *Bacteroides fragilis* directs the development of immunosuppressive Tregs [78]. In addition, SCFA, butyrate and propionate favor the expansion and immunosuppressive activity of Tregs [79]. Amongst microbiota secreted neurotransmitters, glutamate favors Th1-mediated immune responses while γ-aminobutyric acid attenuates Th1 responses and favors Treg activity [80]. It is likely that autoreactive CD4+ T cells, activated after encountering cognate antigens in the gut-associated lymphoid tissues and leading to dysbiosis, promote the acquisition of Teffs, such as Th1 and Th17 [66, 81].

Considerable evidence supports the role of gut microbiota on microglial function and phenotype [67, 82]. Germ-free mice displayed global microglial defects with abundant immature phenotypes [83]. Similarly, native microbiota elimination using antibiotic treatment disrupted microglial maturation evidenced by defective inflammatory gene profiles [84]. Mice exhibiting innate immune cells lacking the free fatty acid receptor 2 (FFAR2) for microbiota’s SCFA also displayed microglial defects. However, recolonization of complex microbiota partially restored microglial defects in germ-free mice [83]. Overall, gut microbiota serves as a clinically feasible target to restore altered innate and adaptive immune responses in different neurodegenerative conditions.

## Dendritic cell role in T cell maturation

The orchestrator of adaptive immune responses is the DC that serves as the body’s key APC participating in immune surveillance and T cell differentiation. Immature DCs encounter antigen through innate pattern recognition receptors (PRRs) such as membrane bound toll-like receptors (TLRs) or cytosolic nucleotide-binding oligomerization domain-like receptors (NLR) and take up antigen by micropinocytosis and phagocytosis. DCs process antigen by proteolytic (endolysosomal and proteosomal) machinery and degrade it into small peptide fragments that bind to major histocompatibility complex (MHC) molecules on the DC surface. The MHC-peptide complexes then present to immunocytes for antigenic-specific stimulations [85, 86]. Although monocyte-macrophages and B cells can also present antigen in a MHC-dependent manner, DCs are unique with the ability to activate naïve T cells and induce antigen-specific immunity [85, 87]. Antigen uptake produces a maturation signal by DCs resulting in upregulation of co-stimulatory molecules like CD40, CD80, and CD86 and secretion of pro-inflammatory signal 3-type cytokines that include IL-6, IL-12, IL-1β, and TNF-α/β [88]. To encounter naïve T cells in the secondary lymphoid organs, DCs upregulate expression of C-C and C-X chemokine receptors on their surface that facilitate their secondary lymph node migration [89]. T cell-DC activation involves a three-signal process. *First*, recognition of cognate antigen on DCs’ MHC by the TCR. *Second*, engagement of the CD28 T cell co-receptor with co-stimulatory molecules CD80/86 on DCs. *Third*, interaction of DC-produced cytokines with their receptors on immunocytes [90]. Upon activation and antigen recognition, naïve T cells differentiate into Teffs facilitating pro-inflammatory immune responses [88, 91].

DCs also maintain central and peripheral immune tolerance. This is attributed to DCs Treg polarizing capability. DCs, termed tolerogenic DCs, can phagocytize self-antigen from apoptotic cells to silence auto-reactive T cells [86]. Tolerogenic DCs display immature or semi-mature phenotypes characterized by low expression of MHC and co-stimulatory molecules and secretion of abundant anti-inflammatory cytokines like IL-10, IL-13, and TGF-β and abrogated pro-inflammatory cytokines. Due to low expression of co-stimulatory molecules, tolerogenic DCs evoke insufficient stimulation signals and therefore differentiate naïve T cells into Tregs instead of Teffs [86, 92]. Both Tregs and Teffs have differential effects on microglia to induce either neuroprotection or neurodegeneration [2, 3] (Fig. 1). The least immune tolerogenic DCs can be induced by immunosuppressive agents and cytokines. IL-10 and TGF-β downregulate MHC and co-stimulatory molecules on DCs, altering their antigen-presentation capabilities and inducing PDL-1 expression and T cell anergy [93]. DCs can also suppress Teff proliferation by immunosuppressive mechanisms shared with Tregs [94]. The immunomodulatory, VIP, attenuates complete maturation of DCs and induces IL-10 secretion [95]. GM-CSF alters expression of co-stimulatory molecules, pro-inflammatory cytokines, and chemokines on DCs that orchestrate immune transformation from Teff into Tregs [96]. Vitamin D3, hepatocyte growth factor, and complement factor H can also induce tolerogenic DCs, which favor Treg polarization [97–99]. Depending upon the state of activation and surrounding microenvironment, DCs favor either Teffs or Tregs to induce antigen-specific immune responses. Thus, DCs mount timed-relevant immune responses by bridging innate and adaptive immune systems [87, 94].

## Effector and regulatory T cells in neurodegenerative disorders

Breaking immunological tolerance through modified or misfolded self-proteins has re-defined the role of autoimmunity in neurodegenerative disorders [3, 100]. CNS draining self-antigens prime peripheral T cells to extravasate inside the brain where T cells are reactivated through cognate antigen recognition and release specific cytokines and chemokines to facilitate extensive local inflammatory reactions. Compromised BBB integrity allows further infiltration of peripheral effector leukocytes [5, 34]. Cytotoxic T lymphocytes (CTLs), T-bet expressing Th1, and RORγ expressing Th17 cells, all Teffs driven neuroinflammatory responses are well-established in various neurodegenerative conditions [1–3]. Several studies have described effects of infiltrating T lymphocytes on microglia and neuronal cells. Teffs (Th1 and Th17) influence and maintain microglia pro-inflammatory phenotypes via secretion of IFN-γ and IL-17 or release of granzyme B [101, 102]. Myelin-specific T cells were observed in direct contact with reactive IL-1β + microglia in the EAE model of MS [103]. Similarly, infiltrating T lymphocytes are found in close proximity of activated microglia with direct neuronal interaction in the substantia nigra (SN) in the MPTP model of PD [1, 13, 29]. In a similar model, overexpression of MHCII on microglia was found after phagocytosis of neuronal tracers to directly interact with infiltrated T lymphocytes [104]. Infiltrated T lymphocytes were observed closely aligned with microglia cells in AD brains [105] where microglial MHCII upregulation corelated with disease progression [106]. The MHCII+ microglia process and present self-antigens to reactivate T lymphocytes that underscore Teff responses on neurodegenerative processes through innate immunity [2]. Like microglia, astrocytes can also interact with T lymphocytes by upregulating MHC and co-stimulatory molecules [107, 108]. Although compared to professional APCs, T cell priming capabilities of astrocytes are relatively weak; hence, direct interaction between astrocytes and reactive T lymphocytes has yet to be confirmed in most neurodegenerative conditions. Studies revealed that IFN-γ stimulated astrocytes cultured from EAE mice upregulated processing and presentation of myelin epitopes to induce Th1 cell differentiation from myelin-specific naïve T cells [109, 110]. Bechmann et al. showed co-localization of astrocytes with T cells in vivo and the ability of astrocytes to induce apoptosis of transformed T cells through the CD95/CD95L pathway [111]. In addition to direct priming T cells, astrocytes may also govern T cell activation and differentiation through instructive cytokines or unidentified molecules including IL-1, IL-6, TNF-α, IL-10, and TGF-β responsible for Teff and Treg differentiation [107, 108]. T cells can also affect astrocyte phenotypes. Th1 secreted IFN-γ has been established as potent stimuli for astrocytes [112]. T cell-derived IL-9 interaction with IL-9R complex on astrocytes was reported to upregulate surface chemokine CCL20 on astrocytes that facilitate Th17 cell transmigration across the BBB [113]. Additionally, direct neurotoxic effects of Teffs have been reported through cell-contact dependent mechanisms and signaling through FasL, LFA-1, and CD40 [114]. Neurotoxicity of Th17 Teffs are well established in MPTP mice [1, 115]. Th17 cells infiltrate into the SN and exacerbate dopaminergic neuronal death through direct contact of leukocyte function-associated antigen (LFA)-1 on Th17 cells with intercellular adhesion molecule-1 (ICAM-1) on neuronal cells. Blocking of either LFA-1 or ICAM-1 using a neutralizing antibody abolished Th17-mediated neurotoxicity, verifying the mechanism of action [115].

In opposite to Teffs, Tregs offer neuroprotective responses. Tregs are a naturally occurring T lymphocyte subpopulation identified by the expression of cell surface markers CD4 and CD25 (IL-2Rα) and the transcription factor forkhead box protein P3 (FOXP3). Tregs, derived thymically or generated peripherally from the naïve T cells upon antigenic stimulation, play an important role in immune homeostasis and antigen-specific immune tolerance by suppressing effector immunity against a divert range of antigens, including those derived from self, commnensal bacteria, and the environment [100, 116]. Tregs downregulate the activities of different immune cells including Teff function and proliferation through several mechanisms. To suppress Teff functions, Tregs release immunosuppressive cytokines including TGF-β, IL-10, and IL-35 or induce direct cytotoxicity and apoptosis by releasing granzyme B and perforin 1. Tregs can also suppress Teff function indirectly through immunoregulatory molecules like cytotoxic T lymphocyte antigen 4 (CTLA4), CD39, and CD73 creating metabolic disruption or by abrogating maturation or antigen-presenting capabilities of APCs. Additionally, as relatively anergic cells, CD25+ (IL-2R) Tregs can adsorb IL-2 from the surrounding environment, concomitantly precluding IL-2 for growth necessitated by CD25+ Th1 and CD8+ Teffs [12, 100, 116].

Emerging evidence suggests alterations in the peripheral adaptive immune system and inflammatory markers in diverse immunological diseases mainly characterized by increased Teff and reduced Treg frequencies or function [5, 9]. However, studies over the past decade warrant that such immune alterations are not limited to the periphery, but also extend to the CNS playing a detrimental role in the development of neurodegenerative disorders [2, 3]. Although Tregs have been characterized in the periphery, recent advancements have enabled study of this tiny population inside the brain as well [33, 48]. Tregs exhibiting an activated memory phenotype have been identified in the brain. This phenotype helps Tregs to suppress local effector immune responses and ameliorate neuroinflammation [116, 117]. During the initial disease stage, the compromised BBB further allows the entry of T lymphocytes, including Tregs, that contribute to CNS immunity [116]. However, over disease progression, the frequency and immunosuppressive properties of Tregs are diminished, shifting balance between Tregs and Teffs, specifically towards IFN-γ producing Th1 and IL-17 producing Th17 cells, leading to a breakdown of immune tolerance [3, 118]. In addition to suppressing the function and proliferation of Teffs, Tregs maintain anti-inflammatory microglial and astrocyte phenotypes through the release of IL-4, IL-10, and TGF-β [12, 54, 119]. Tregs suppress microglial synthesis and release of ROS, and facilitate astrocyte glial cell-derived neurotrophic factor (GDNF) and brain-derived neurotrophic factor (BDNF) secretion [22, 29]. Tregs also protect neurons directly using cell-contact dependent mechanisms via CD47 and signal regulatory protein-α (SIRPA) [120] or by releasing neurotrophic factors such as BDNF [12, 52]. Neuronal cells can also interact directly with microglia via the CD200-CD200R mechanism [54, 121]. Neuronal ligand CD200 interact with microglia receptor CD200R to induce an anti-inflammatory microglial phenotype that attracts Tregs by release of chemokines, possibly C-C chemokine ligand 2 (CCL2) and CCL5 [54, 122].

Tregs also have regenerative activities in tissues that include the retina [123], kidney [124], and skin [125]. This is seen through the control of tissue-specific inflammatory responses as well as through direct regenerative responses. Indeed, depletion of Tregs has been shown to augment tissue damage and mortality and affect vascular repair in these tissues. Although reparative functions of Tregs are not fully characterized in the brain due to their lower frequency, recent advancements have established functional role of Tregs in brain repair. The regenerative potential of Tregs was found through direct differentiation of oligodendrocyte progenitor cells that promote remyelination in the absence and presence of overt inflammation. Treg-derived cellular communication network factor 3 (CCN3), a growth regulatory protein, was identified as a key driver of oligoprogenitor cell differentiation and CNS remyelination [18]. Recently, brain reparative potential of peripheral Tregs was seen in ischemic stroke. In chronic disease phase, infiltration of peripheral Tregs increased in the ischemic mouse brain that efficiently suppressed neurotoxic astrogliosis by producing amphiregulin, a low-affinity epidermal growth factor receptor ligand that induced neuronal recovery from ischemic inflammatory responses [119]. However, these findings do not rule out additional indirect mechanisms of Treg neuroprotection and repair.

Both Tregs and Teffs are crucial in maintaining systemic as well as central immune homeostasis; therefore, preservation of these immune cell frequency and function can control neurological diseases [2, 3] (Fig. 2). Several studies have shown the peripheral emergence of Teffs followed by their infiltration into the CNS with concurrent downregulation of Treg function and frequency in different neurodegenerative disorders [1, 126]. Here, we have summarized the effects of Treg-Teff imbalance in several diseases and how restoring its balance can improve conditions.

### Multiple sclerosis (MS)

CNS-antigen specific immune responses were first identified in MS patients and in EAE animals. The prime etiology of this autoimmune disease is loss of self-tolerance that allows development of functional autoreactive lymphocytes against their cognate antigen [2, 3]. In attempt to decrease autoreactive cells, MS patients were injected with modified self-peptide. Surprisingly, treatment enhanced autoreactive Th1 responses and triggered development of newer inflammatory lesions. This clinical trial outcome provided evidence of T cell epitope spreading and their direct role in CNS defects in MS [127]. In MS, disease specific Teff frequency increases in the periphery that subsequently infiltrate into the CNS. MS patients showed increased peripheral Th1 and Th17 cells along with their associated cytokines including IL-1β, IL-6, IL-17, TNF-α and IFN-γ [34, 128]. Increased frequencies of myelin basic protein (MBP)- and myelin proteolipid protein (PLP)-reactive CD4+ and CD8+ T lymphocytes were observed in the peripheral blood and CSF of MS patients. IL-2 stimulation further increased autoreactive CD4+ T cell frequency in MS patients [129].

Additionally, autoreactive CD4+ and CD8+ T cells were found in post-mortem diseased brains but not in healthy controls [130]. In the CSF and brain parenchyma of MS patients, IFN-γ, IL-17 and IL-22 were elevated compared to controls. Activated CD4+ T cells simultaneously expressing IFN-γ and IL-17 were predominantly expanded in MS patients’ blood during relapse phase. This study demonstrated a greater capacity of these cells to invade the BBB as evidenced by histopathological examination of post-mortem MS brain tissues [131]. In EAE models of human disease, adaptive immune responses can be induced either by subcutaneous immunization with myelin antigens or by adoptive transfer of in vitro myelin-activated CD4+ T cell subsets, mainly consisting of IFN-γ-producing Th1 cells and IL-17-producing Th17 cells [132, 133]. In rodents, immunization with MBP peptides activated myelin-specific T cells in the periphery with concomitant infiltration of T lymphocytes into the brain. Infiltrated Teffs, specifically Th1 and Th17, accumulated in the brain parenchyma and initiated demyelination. Likewise, the adoptive transfer of induced Teffs progressed disease in the naïve recipient [134]. Adoptive transfer of encephalitogenic Th1 and Th17 cells induced CNS lesions with distinct patterns [135, 136]. The predominance of Th1 or Th17 responses in MS patients has been implicated in disease heterogeneity with variation in clinical scores, responses to immune therapies, and localization of CNS lesions [137, 138]. Thus, studies demonstrated extravasation of peripheral Teffs into the CNS that orchestrate autoimmune neuroinflammatory responses in EAE and MS. Although the key role of Th1 and Th17 Teffs in the progress of MS and EAE are well established, the mechanisms whereby these cells contribute to the pathogenesis are not completely understood. The possible mechanism of breakdown of self-tolerance might be attributed to Teff induced altered Treg frequencies and function [2, 3].

Contrary to increased Teff frequency, the number, function, and migratory capabilities of Treg are compromised in the MS patients. Although significant differences in the number of circulating Tregs between MS patients and healthy controls are not always observed, isolated Tregs have lower suppressive activity as frequently reported, suggesting a functional Treg deficit contributes to a breakdown of immune tolerance in MS [139, 140]. A recent meta-analysis study demonstrated that the frequency of peripheral Treg defined as CD4+CD25+FOXP3+ significantly decreased in MS patients compared to control subjects [141]. No differences were observed when Tregs, defined by only CD4+CD25+, suggesting the importance of FOXP3 as a vital Treg marker that plays a key role in maintaining immunosuppressive functions. Restoring Treg frequency and function has shown promising results in disease outcomes. IL-10 gene therapy in EAE significantly increased Treg frequency by modulating DCs. This resulted in improved clinical symptoms and attenuated gliosis in the brain [142] possibly through IL-10 induced DC tolerance that has the ability to increase Treg frequency and function. Treg depletion attenuated the protective effects offered by intravenous immunoglobulin (IVIg) in EAE [143]. Furthermore, VIP administration expanded Tregs in the periphery and the CNS and simultaneously inhibited encephalitogenic T cell activation and abrogated disease progression [144]. Of note, VIP is a potent inducer of tolerogenic DCs which can increase Treg frequency and function in a cytokine-dependent manner [95]. Fingolimod, an immunomodulator that inhibits CNS ingress of lymphocytes from lymph nodes and their recirculation and is approved for the treatment of MS, has also been reported to reduce circulating IFN-γ and IL-17 secreting Teffs and increase the number of circulating Tregs in MS patients [145, 146].

Over the past decades, our understanding of neuroinflammation has increased beyond its role in classical autoimmune diseases like MS. Research efforts have now established the role of adaptive immune alterations (Tregs and Teffs) in the pathogenesis of classical neurodegenerative disorders including AD, PD and ALS, and related vascular and viral-based neuronal injuries, stroke and HIV-1 associated neurocognitive disorders (HAND) respectively.

### Alzheimer’s disease (AD)

**Fig. 3:**
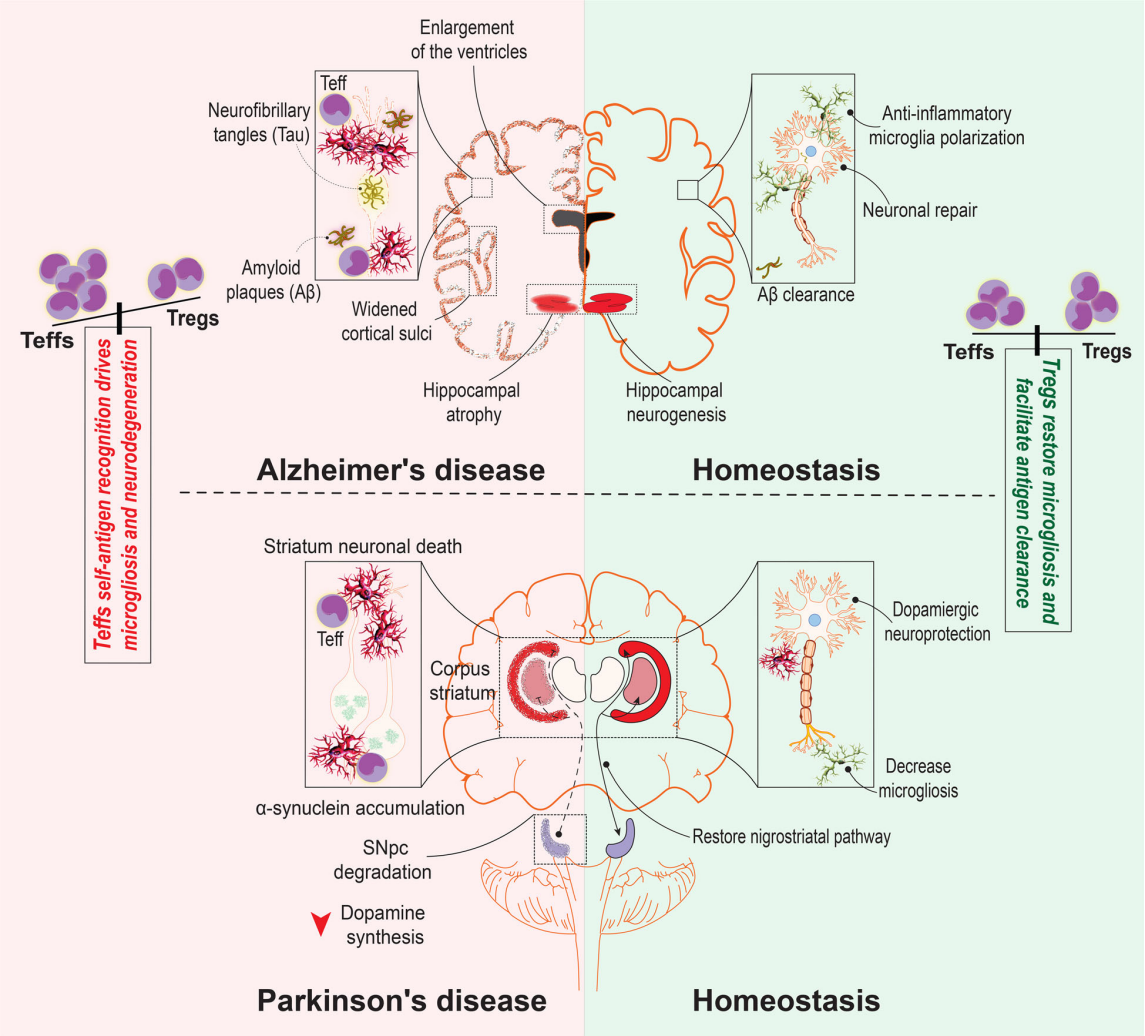
Teff and Treg immunity in AD and PD. Pathogenic changes observed in AD brain include accumulation of intraneuronal neurofibrillary tangles (NFTs) of Tau protein and extracellular amyloid beta (Aβ) plaques. The accumulated peptides facilitate neuroinflammatory Teff entry into the brain where they affect resident microglia cells to induce local inflammatory responses. In PD, α-synuclein accumulation promotes immunoreactive Teff entry into the brain to activate microglia, compromising nigrostriatal axis between the substantia nigra pars compacta (SNpc) and striatum that execute motor commands. Immunoregulatory Tregs can restore homeostatic balance in the brain through clearance of neuroimmunogens, microglial polarization and restoration of normal neural functions

AD is the most common cause of dementia in the elderly. AD affects an estimated 40 million people worldwide, and this is expected to double every 20 years until at least 2050 [147]. The disease is mainly characterized by the extracellular deposits of Aβ plaque and intraneuronal neurofibrillary tangles (NFTs) of tau triggering degeneration of cholinergic neurons in the brain (Fig. 3) [17, 148]. Although most AD research is progressing around the amyloid hypothesis, recent findings have argued the role of sustained inflammation secondary to misfolded protein accumulation that affect microglia and neuronal cell functions. Innate and adaptive immune alterations play a key role in driving such neuroinflammatory responses [2, 3, 17]. Active Aβ immunization showed promising results in preclinical testing that abrogated amyloid deposits and improved cognitive functions [149]. However, a phase 2a clinical trial (NCT00021723) testing immunization with full length Aβ and QS21 adjuvant (AN1792) was paused after the observation of meningoencephalitis in 6% of patients. Later, the adverse effects were found to be attributed to the pro-inflammatory T lymphocyte infiltration into the brain parenchyma [150]. In contrast, Aβ-specific CD4+ T cells have shown to promote Aβ clearance and reverse cognitive decline in AD mouse models [151, 152]. Therefore, CD4+ T cells can be detrimental or protective depending upon their effector or regulatory phenotype.

Increased frequency of circulating Teffs and their subsequent transmigration into the brain parenchyma has been observed in AD patients and murine models [105, 126]. During AD progression, brain amyloidosis alters the expression of vascular adhesion molecules that enhance the transmigration of peripheral immune cells into the brain [153]. Increased numbers of infiltrating T cells were observed in amyloid-burdened brain regions of AD transgenic mice with concomitant up-regulation of endothelial adhesion molecules ICAM-1 and vascular cell adhesion molecule-1 (VCAM-1) compared to non-transgenic littermates. Infiltrated T cells do not proliferate locally nor release less IFN-γ, but interfere with local antigen presentation and T cell activation, suggesting their inability to contribute protective immunity in the amyloidogenic brain [154].

It was also shown that *Bordetella pertussis* respiratory infection amplified migration of IFN-γ- and IL-17-producing T cells and NK T cells in the brain of older human amyloid precursor protein (APP) and presenilin 1 (PS1) double transgenic (APP/PS1) mice. Later, this process was confirmed to be age-dependent and, showed significantly higher numbers of Th1 and Th17 cells in older APP/PS1 mice with parallel gliosis [155]. Common infectious pathogens that include *cytomegalovirus*, *herpes simplex virus type 1*, *Borrelia burgdorferi*, *Chlamydophila pneumoniae* and *Helicobacter pylori* were found associated with elevated systemic inflammation and amyloid burden in AD patients [156, 157]. Chronic infection with these agents also developed cerebrovascular disorders [158] that subsequently promoted AD pathology [159] in patients. Thus, chronic infection and persistent peripheral inflammation may be associated with increased T lymphocyte migration into the brain that lead to autoimmune neurodegeneration.

Brown et al. observed significant infiltration of IFN-γ- and IL-17-secreting T lymphocytes in APP/PS1 mice brain. Additionally, adoptive transfer of Aβ-specific Th1 cells, but not Th2 and Th17 cells, increased microglia activation and amyloid deposition that led to early cognitive impairment in mice [160] suggesting a key role of antigen-specific Teff responses in propagating an inflammatory cascade to further disease pathology. Interestingly, both IFN-γ-secreting CD4+ as well as CD8+ T lymphocytes were detected in the CNS following adoptive transfer of Aβ-Th1 cells reflecting a key role of both T cell subsets in AD progression. Recently CD4+ and CD8+ lymphocytes were found patrolling the CSF in AD patients with clonal expansion of CD8+ Teffs [161, 162]. Extravasation of CD4+ and CD8+ Teffs was observed in the brains of postmortem AD patients compared to controls who died of non-neurological diseases [105, 126, 161, 163]. Overall, a clear association of adaptive immunity in the neuropathogenesis of AD was demonstrated.

Apart from CNS, peripheral immune alterations are likely candidates for early biomarker studies in different neurodegenerative diseases including AD. Peripheral blood analysis of AD patients showed elevated frequencies of CD4+ and CD8+ T lymphocytes [161, 162]. Saresella et al. first observed elevated levels of RORγ+ Th17 cells in AD patients’ blood compared to those with mild cognitive impairments (MCI) and age-matched controls. However, Th1-associated transcription factor T-bet and cytokines were unchanged. Despite increased effector memory and terminally differentiated lymphocytes in both disease patients (AD and MCI), authors selectively identified peripheral Th17 cell activation as an important immunological marker to differentiate AD and MCI patients [164]. Later Agnes et al. showed increased proportion of Th17 cells and their direct involvement in the systemic inflammatory functions in AD patients [165] further supporting Th17 Teffs as a potential biomarker. Peripheral blood IL-17, a key Th17 cell cytokine, can weaken tight junctions of the BBB and facilitate peripheral leukocytes extravasation into the brain inducing pro-inflammatory cytokines TNF-α, IL-1β, and IL-6 in AD [166, 167]. In line with previous reports, Oberstein et al. recently suggested an early role of peripheral Teffs in disease progression. Authors observed increased circulating Th17 cells in early stage AD patients exhibiting MCI that correlated to amyloidopathy [168]. Another study also revealed direct Aβ effects on Th17 cells cytokine expression [169]. Intrahippocampal Aβ_1–42_ injection in rats disrupted BBB integrity that allowed peripheral RORγ+ Th17 cell infiltration at the injection site as evidenced by elevated IL-17 and IL-22 expression in the hippocampus. These cytokine levels were also elevated in CSF and serum. Additionally, infiltrated Th17 cells induced direct neurotoxicity via the Fas-FasL apoptotic pathway. With elevated circulating CD4+ Teff frequency, downregulated Treg frequency was also reported [170].

The idea of counteracting effector immune responses using Tregs is still controversial in AD pathogenesis. Naturally occurring Tregs were reported to interfere with ability of retinal ganglion cells to counterattack aggregated Aβ toxicity and depletion of Tregs helped revitalize retinal neuronal tissue [171]. Increased Treg frequency and their suppressive function in aging AD patients were also reported [172]. Treg immunosuppression impaired phagocytic capabilities of brain resident innate immune cells and affected amyloid plaque clearance in an AD mouse model [173]. Despite these facts, most evidences support the protective effects of Tregs in AD. Recently, Ciccocioppo et al. reported decreased Treg frequency in AD and MS patients of a similar pattern [174] suggesting common roles of impaired Treg immunological tolerance in both the diseases. Dansokho et al. demonstrated that peripheral Treg depletion accelerated cognitive impairment in AD mice without affecting amyloid deposition. Such early cognitive impairment was linked with microglia activation and altered disease-related gene expression profile [175]. Beak et al. showed that Treg depletion markedly increased amyloid plaque deposition and aggravated spatial learning abilities in 3xTg-AD mice. Adoptive transfer of Tregs significantly corrected cognitive deficits and reduced amyloid burden, while Teff transplantation worsened the behavioral outcome in recipients [23]. Dansokho et al. and Beak et al. reported detrimental effects of Treg depletion on memory and microglial function [23, 175]. In contrast, Baruch et al. reported amyloid clearance and improved cognition following Treg depletion in AD mice [173]. The major difference amongst studies that earlier studies were performed using early age diseased mice when amyloid deposition and gliosis have just started; while in the later study, researchers depleted Treg at an intermittent disease stage when cerebral Aβ plaque and gliosis have significantly developed. Additionally, different strategies of Treg depletion were employed in studies. Importantly, Baruch et al. results suggested that transient Treg depletion promotes late recruitment of immunoregulatory Tregs and monocyte-derived macrophages, which play a role in mitigating neuroinflammatory responses [173]. In another study human umbilical cord-derived mesenchymal stem cells (hUC-MSCs) treatment of Tregs from APP/PS1 mice restored their immunosuppressive function and their transplantation significantly reduced Aβ plaque deposition and corrected cognitive impairments in recipient mice. The results were accomplished by reduced IFN-γ, increased TGF-β1 and IL-10 levels in the periphery, and attenuated brain microgliosis [176]. Together, these reports support neuroprotective capabilities of systemic Tregs in AD pathogenesis attributed to their cytokine dependent direct effects or indirect effects on CNS innate immune cell function.

Therapeutic approaches to boost peripheral Treg frequency and function can be harnessed for effective AD treatment. For instance, amplification of Tregs with low dose IL-2 increased number of plaque-associated microglia, reduced Aβ load, and restored cognition in APP/PS1 mice [175]. Later, reduced IL-2 levels were observed in the hippocampal biopsies of AD patients. A single AAV-IL2 injection induced prolonged Treg expansion and activation in both periphery and brain of APP/PS1 mice. In the hippocampus, IL-2 activated astrocytes surrounding amyloid plaques and improved synaptic plasticity to recover memory deficits [177]. Together, studies propose adjunct IL-2 in immune modulatory therapeutic regimens of AD patients to boost Treg frequency and function. Treatment of bee venom phospholipase A2 (bvPLA2) significantly improved cognitive function and reduced hippocampal Aβ deposits in 3xTg mice. Effects were attributed to Treg induction because Treg depletion using anti-CD25 antibody abrogated bvPLA2 neuroprotective effects [178]. Fingolimod reduced amyloid burden and microglia activation in 5xFAD mice by promoting Tregs entry inside the CNS with simultaneously attenuating other Teffs entry by sequestering them into the peripheral lymph nodes [179]. Similar anti-inflammatory mechanism of fingolimod has been reported in MS patients [145] and a traumatic brain injury model [180]. Recently, our laboratory demonstrated immunomodulatory effects of GM-CSF. GM-CSF treatment induced Treg frequency in the periphery that ameliorated amyloid burden and microglia activation in APP/PS1 mice brains. Improved behavioral function was also attributed to its Treg effects [17]. Likewise, a recent phase II clinical trial utilizing sargramostim [Leukine®, recombinant human GM-CSF (rhGM-CSF)] demonstrated significant changes in mini-mental state examination (MMSE) scores in mild-to-moderate AD patients [181]. Although the study did not evaluate Treg populations, improved memory outcome in AD patients might be attributed to GM-CSF Treg effects that can be studied in future. The overall differential effects of Teffs and Tregs in AD pathogenesis are illustrated in Fig. 3.

### Parkinson’s disease (PD)

Following AD, PD is the second most common neurodegenerative disease associated with progressive loss of motor function. Available treatments affect symptoms, but do not alter disease progression. PD is classically characterized by the loss of dopaminergic neurons within the substantia nigra (SN) along with their projections into the striatum resulting in insufficient dopamine release that leads to progressive motor impairment. The remaining surviving neurons often contain intracellular inclusions, called Lewy bodies. These are composed of modified α-synuclein and are associated with microgliosis which serve as a key pathological hallmark of the disease (Fig. 3) evidenced only after post-mortem analysis of the patient brain [3]. Considerable evidence shows that infiltration of peripheral immune cells and microglial activation are key mediators of the persistent neuroinflammatory responses in PD that subsequently lead to dopaminergic neuronal cell death [13]. Studies have demonstrated the presence of CD4+ and CD8+ T cells in the SN of post-mortem brains from PD patients. Similarly, T lymphocyte infiltration was observed in the MPTP mouse model of PD, where T cells were associated with nigrostriatal neuronal cell death [1, 13, 29, 182]. CNS infiltration of T lymphocytes occurs following their early peripheral activation; therefore, altered T lymphocyte populations in PD patients’ blood could likely be considered for early disease diagnosis. Several studies have revealed alterations in peripheral immune subsets in PD patients [24, 183, 184]. Previously, our laboratory demonstrated the association of motor severity with peripheral T lymphocytes in PD patients; wherein, increased effector memory T cell (Teff) frequency was associated with impaired motor function [118]. Although no differences in Treg and Teff frequencies between PD patients and caregivers were observed, the immunosuppressive capability of Tregs was compromised in PD patients and highlights disease associated functional Treg deficits. Baba et al. reported decreased CD4+/CD8+ T cell ratio and CD4+CD25+ Treg frequency in PD patients compared to healthy controls. In PD patients, a phenotypic shift towards Th1-type immune responses was observed due to IFN-γ producing Th1 cells were significantly upregulated compared to IL-4 producing Th2 cells [185]. In this study, patients treated with levodopa showed significantly lower number of different T cell subsets compared to controls. However, Bas et al. reported a similar reduction in lymphocyte count of PD patients irrespective of Levodopa treatment [186] suggesting that lymphocyte alterations are not directly dependent on dopaminergic treatment. Other studies showed elevated frequencies of Th1 [184] and Th17 [187] Teffs in the peripheral blood of PD patients with concurrent occurrence of T lymphocytes in postmortem brain specimens [13]. Chen et al. suggested the active role of peripheral immune system in the progression of PD. Using PD patients’ blood analyses, authors concluded the presence of increased proportions of Th1 and Th17 cells with decreased Th2 and Treg subtypes. The elevated Th1/Th2 ratio was associated with motor function scores determined by Unified Parkinson’s Disease Rating Scale-III (UPDRS-III), but not with elevated Th17/Treg ratio. This may be because UPDRS-III may not fully reflect the disease severity [183]. In cross-sectional studies, Kustrimovic et al. observed reduced circulating CD4+ T cells in PD patients that included Th2, Th17, and Treg populations [184]. Isolated naïve CD4+ T cells preferentially differentiated towards the Th1 lineage in both PD patients (drug-naïve and dopaminergic drug treated) further verified that current symptomatic treatments are unable to modulate ongoing peripheral adaptive immune alterations. Overall, studies suggest effector adaptive immune alterations are operative over the disease course and may serve as novel biomarkers in PD diagnosis. In the brain, dopaminergic neuronal cell death releases modified protein α-synuclein into the extraneuronal environment causing activation of resident microglia and subsequent induction of antigen-specific Teff populations in the secondary lymphoid organs [2, 3]. Thus, it is likely that disease-causing Teffs are initially activated in the periphery where they undergo clonal differentiation and expansion in order to enter the PD brain.

Tregs can be harnessed to attenuate such effector immune responses to maintain central and peripheral immune tolerance and diminish neuroinflammation [188]. Tregs not only suppress effector immune responses but also transform neurodestructive Th1 and Th17 responses into neuroprotective ones [1], suggesting immunomodulator potential of Tregs in PD (Fig. 3**).** Depletion of Tregs can exacerbate neuroinflammation in the MPTP mouse model of PD [189] suggesting their direct role in neuroprotection. Direct protective effects of Tregs on MPTP-intoxicated primary ventral mesencephalic dopaminergic neurons was demonstrated. It was shown that CD47-SIRPA interactions was the mechanism independent of Treg associated TGF-β1 and IL-10 [120]. Interaction of CD47 on Treg with SIRPA on dopaminergic neuronal cell activated the neuronal Rac1/Akt signaling pathway to mediate the observed neuroprotection. Previously, our own laboratory demonstrated that adoptive transfer of CD4+ T cells isolated from copolymer-1 (glatiramer acetate) immunized mice to MPTP-intoxicated recipient mice significantly protected the nigrostriatal dopaminergic system by attenuating microglial activation [29]. The results were likely attributed to the Treg responses as copolymer-1 is a potent inducer of this cell type [28, 190, 191]. Later, our laboratory adoptively transferred CD3-activated CD4+CD25+ Tregs into MPTP-intoxicated mice. A dose-dependent neuroprotective response was observed with concomitant suppression of microglia activation, leading to increased survival of dopaminergic neurons in the SN [22]. Adoptive transfer of T cells from mice imminzed with nitrated alpha-synuclein (N-α-synuclein} to MPTP-treated mice exacerbated neuroinflammation and nigrostriatal degeneration, which was attributed to Th1 and Th17 mediated immune responses with parallel Treg dysfunction [1, 16]. Adoptive transfer of natural Tregs or Tregs induced by VIP significantly attenuated microglial inflammatory responses leading to robust nigrostriatal neuroprotection in MPTP mice. Thus, we described the therapeutic implications of peripheral Treg induction in PD. Later to overcome the limitations of native VIP for clinical application, we developed a selective VIP receptor 2 (VIPR2) agonist that reduced IL-17A, IFN-γ, and IL-6 release and increased GM-CSF transcripts in CD4+ T cells, induced phenotypic shift towards Treg [25]. Our laboratory also has used recombinant GM-CSF to increase Treg numbers and function in PD patients and animal models. In the MPTP mice model, GM-CSF induced Treg number and function leading to nigral dopaminergic neuroprotection and corrected microglia responses. The results were confirmed when adoptive transfer of GM-CSF-induced Tregs showed nigral neuroprotection in recipient MPTP mice [26]. Moreover, in a phase I clinical trial, sargramostim (rhGM-CSF) treatment improved UPDRS-III scores and magnetoencephalography-recorded cortical motor activities in PD patients. GM-CSF treatment increased Treg frequency and function that contributed protective effects in patients [24]. As discussed, both VIP and GM-CSF are potent inducers of tolerogenic DCs that could be a possible mechanism of Treg induction [95, 96]. Direct neuroprotective effects of GM-CSF were also reported. GM-CSF protected MPP+ treated PC12 cells and mouse primary mesencephalic neurons in vitro by modulating apoptosis related proteins. The results were confirmed in vivo where GM-CSF protected dopaminergic neurons in the SN and improved locomotor activity in MPTP mice [192]. Overall studies suggest direct as well as indirect neuroprotective mechanism of GM-CSF in PD. Ginsenoside Rg1 treatment abrogated peripheral and central inflammation in MPTP mice. Rg1 protected dopaminergic neurons by inhibiting microglia activation and CD3+ T cell infiltration into the substantia nigra pars compacta (SNpc). In the periphery, Rg1 increased FOXP3+ Tregs while reducing inflammatory cytokines TNF-α, IFN-γ, IL-6, and IL-1β [193]. Although the anti-inflammatory property of Rg1 has been reported in the neurological diseases [194], its immunoprotective mechanisms seek further investigation. bvPLA2 can also induce peripheral Tregs to promote dopaminergic neuronal survival in MPTP mice. The protective effects were associated with microglia deactivation and limited CD4+ T cell infiltration into the brain. Interestingly, the protective effects of bvPLA2 were reversed upon depletion of Tregs. bvPLA2 directly bound to CD206 on DCs and constitutively promoted the secretion of PGE2 that via PGE2 (EP2) receptor signaling facilitated Treg differentiation from FOXP3-CD4+ T cells [195]. The finding further highlights the key role of DCs in immunoprotection by transforming Tregs from Teffs. Taken together, these findings suggest that therapeutic approaches to amplify peripheral Treg frequency and function would be promising in clinics over conventional symptomatic treatments. Different immunomodulators with the ability to increase peripheral Treg frequency and function and their downstream neuroprotective effects are summarized in Table 1.

**Table 1:** Peripheral Treg expansion for disease combating neuroprotection in different neurodegenerative animal models

Treg expansion	Disease	Experimental model	Effects
Adoptive transfer	MS	EAE	Resistance to reinduction of EAE [196]. Mice are disease-free [197].
	AD	3xTg	Reduced Aβ plaque deposition and improved behavior [23]
	PD	MPTP	Attenuated Th17 neurodestructive and microglial inflammatory responses and induced nigrostriatal protection [1]
	ALS	mSOD1/RAG2^−/−^, mSOD1^G93^	Tregs isolated from disease mice prolonged survival [21, 198, 199]
	Stroke	MCAO	Reduced brain infarction [200], attenuated inflammation and BBB damage [201]. Exerted early neuroprotection without entering the brain [202]. Promotion of neurogenesis [203] and remyelination [18].
Low dose IL-2	MS	EAE	Pre-treatment only attenuated EAE [204].
	AD	APP/PS1, APP/PS1ΔE9	Restored cognitive function, increased number of plaque associated microglia [175] and astrocytes [177]
GM-CSF	AD	APP/PS1	Increased Aβ clearance and improved cognition. Recruitment of microglia surrounding Aβ plaque, improved synaptic plasticity and neurogenesis [17]
	PD	MPTP	Protected tyrosine hydroxylase immunoreactive (TH+) neurons in SN, attenuated microglial activation and improved motor functions [26, 192]
Vasoactive intestinal peptide (VIP)	MS	EAE	Inhibited encephalitogenic T cell activation and slowed disease [144]
	PD	MPTP	Attenuated microglial activation and spared TH+neurons in SN. Phenotypic shift of effector cells to Treg was observed [25]
Fingolimod	MS	EAE	Inhibited peripheral Teffs entry inside the CNS by sequestering them into lymph nodes but allowed Tregs entry [145, 146]
	AD	5xFAD	Decreased amyloid plaque and microglia activation and promoted anti-inflammatory neuroprotective responses [179]
IL-2/IL-2 antibody complex (IL-2/IL-2Ab)	MS	EAE	Development of resistance to induction of EAE [205] and reduced disease severity [206]
	ALS	mSOD1^G93A^	Slowed down disease progression rate and increased survival period [207]
	Stroke	MCAO	Early Treg protective effects independent to their brain penetration by suppressing peripheral Teffs. Also attenuated central neuroinflammation and protected against brain injury [208].
	Traumatic brain injury (TBI)	controlled cortical impact (CCI)	Attenuated neutrophil infiltration and inflammation leads to improved neurological recovery [209].
Bee venom phospholipase A2	AD	3xTg	Decreased Aβ deposits in hippocampus and enhanced cognitive function. Microglia deactivation and reduced CD4+ T cell infiltration [178, 210]
	PD	MPTP	Induced microglia deactivation and attenuated CD4+ T cell infiltration [189, 195, 211]
Ginsenoside Rg1	PD	MPTP	Inhibited microglia activation and CD3+ T cell infiltration [193]
Intravenous immunoglobulin (IVIg)	MS	EAE	Prevented CNS infiltration of Teffs and almost completely protected mice from EAE [143]
Atorvastatin	Stroke	MCAO	Prevented infarct and glia activation [212]

### Amyotrophic lateral sclerosis (ALS)

**Fig. 4:**
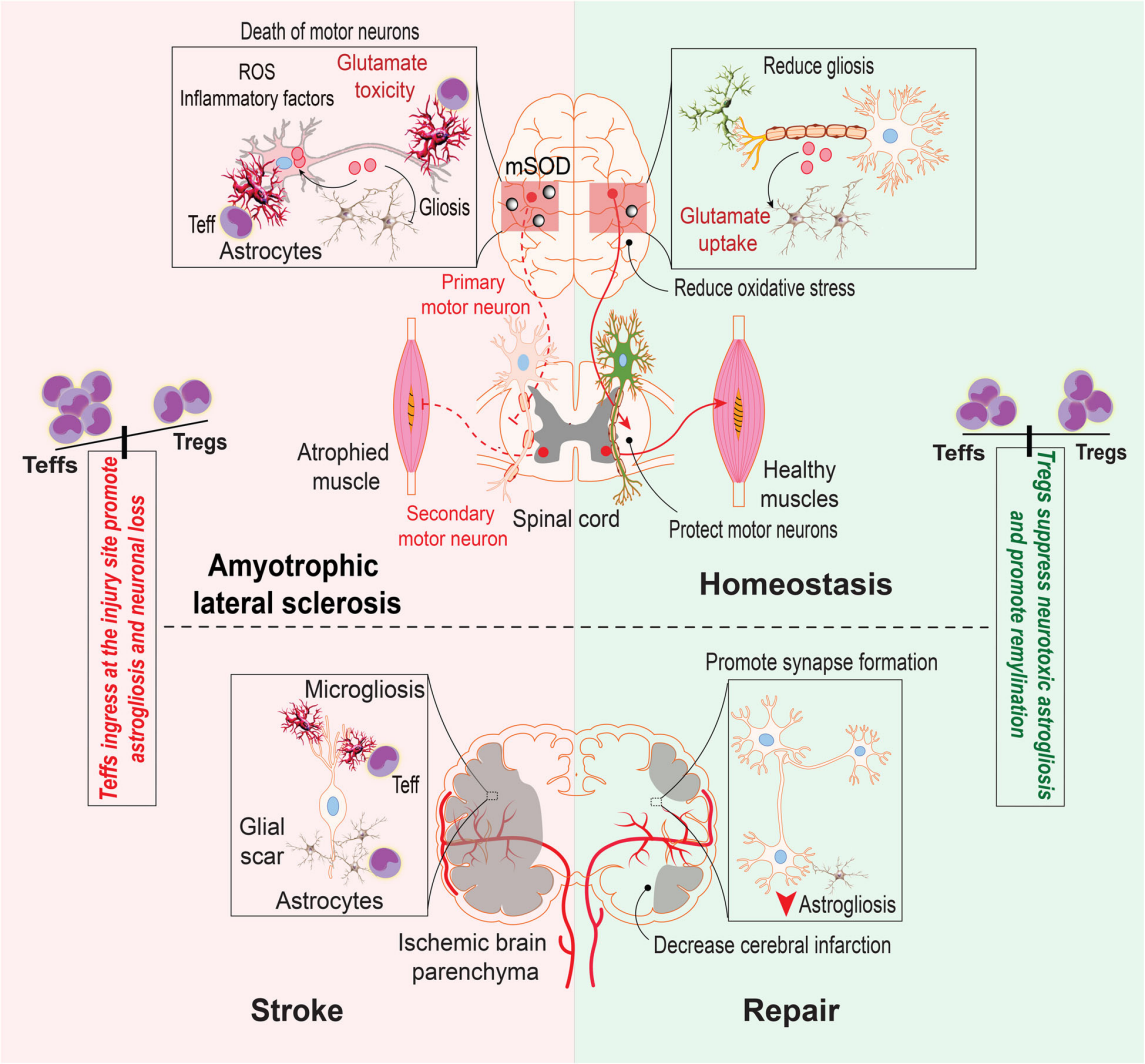
Teff and Treg immunity in ALS and stroke. In ALS, Teffs perpetrate innate microglial inflammation by misfolded SOD inciting oxidative stress and affecting astrocyte function linked to glutamate uptake, ensuring motor neuronal cell death with the primary clinical manifestations of disease. Parallel responses are operative in Stroke. Following ischemic stroke, peripheral Teffs accumulate at the brain injury site to participate in local inflammatory responses manifested by micro- and astrogliosis and secondary neuronal injuries. Following acute episode, injuries are contained, in part, through the emergence of Tregs that serve to reduce astrogliosis, promote synapse formation, and decrease the extent of injury. Tregs are neuroprotective mediators in each of these pathological processes and herald slow disease progression and control of neurodegenerative activities

ALS is a progressive neurodegenerative disease with unknown etiology primarily affecting upper and lower motor neurons in the motor cortex, brain stem, and spinal cord. ALS pathogenesis mechanisms include glutamate excitotoxicity and dominant mutations in the gene for superoxide dismutase 1 (SOD1) leading to mitochondrial toxicity. This facilitates recruitment of astrocyte and microglia cells surrounding damaged motor neurons in the spinal cord and brain, triggering oxidative stress by releasing ROS and other pro-inflammatory mediators to orchestrate motor neurodegeneration (Fig. 4) [213, 214]. ALS pathogenesis consists of mainly two stages: an early, slow progressive neuroprotective stage and later, a rapidly progressing neurotoxic stage associated with anti-inflammatory and pro-inflammatory immune responses, respectively [213, 215]. Neuroinflammation is a common feature among multifactorial disease etiologies characterized by the alterations in innate and adaptive immune responses in both periphery and CNS [214, 215]. Studies over the past decades have demonstrated the infiltration of peripheral T lymphocytes and APCs that secrete a variety of cytokines in the spinal cord and brain of ALS patients. Troost et al. first identified substantial infiltration of T cells (including CD4+ and CD8+) and macrophages in the corticospinal tracts and anterior horn of the spinal cord in ALS patients [216]. Such infiltrations were observed secondary to spinal cord atrophy suggesting activation of peripheral adaptive immune system follows initial neurodegeneration. Later, Kawamata et al. identified CD4+ and CD8+ T lymphocyte migration along the capillaries and venules of the pre-central gyrus in ALS patients that extended up to the parenchyma exhibiting neuronal damage [217]. The study further confirmed autoimmune alterations inside the brain and spinal cord of ALS patients secondary to neuronal demise. Engelhardt et al. found perivascular and intraparenchymal T lymphocyte infiltration in the corticospinal tract and ventral horns of the spinal cord during autopsies of ALS patients [218]. Graves et al. reported perivascular infiltration of T lymphocytes along with macrophages and mast cells in the spinal cord and brain of ALS patients [219] suggesting both adaptive and innate immune systems are activated over the disease course. Similar CD4+ and CD8+ T lymphocyte infiltration was observed and associated with microglia activation in the brains of ALS mice [220]. Studies suggest that effector immune responses are extended into the CNS from the periphery where they contribute to systemic inflammation. Saresella et al. demonstrated increased expression of both Th1 and Th17 Teffs with reduced frequency of IL-10 and TGF-β expressing Tregs in the peripheral blood of ALS patients comparable to MS patients [221] suggesting common systemic inflammatory immune responses between ALS and MS. Rentzos et al. identified elevated frequency of CTLs and NK T cells with simultaneous reduction of Tregs in peripheral blood of ALS patients where Treg frequency negatively correlated with the disease progression rate [222], suggesting the indisputable role of impaired Treg suppressive circuit in ALS immune activation.

Tregs increase during the early slow progressive disease stage, protecting M2 microglia while decreasing during the later rapidly accelerating disease stage [215]. Beers et al. demonstrated that passive transfer of endogenous Tregs from early disease staged mutant SOD1 (mSOD1) mice into advanced disease mice sustained IL-4 expression and M2 microglia phenotype leading to extended stable disease stage in recipients. The results were extended to ALS patients and showed reduced Treg frequency in rapidly progressing patients [198]. Thus, the study suggested a beneficial role of expanding peripheral Tregs to suppress toxic neuroinflammation in the CNS of ALS patients. During the rapidly progressing disease phase, decreased peripheral Treg frequency in mSOD mice paralleled with increased proliferation of Teff, opposite to early slow disease phase. Therefore, systemic transplantation of Tregs isolated during the slow phase suppressed Teffs and microglial neurotoxicity during rapid progressive phase [21]. Decreased Treg number and FOXP3 expression were found in rapidly progressing ALS patients suggesting functional Treg deficits over disease period as FOXP3 transcription factor is a key determinant of Treg immunosuppressive function. The expression of TGF-β, IL-4, and GATA-3, a Th2 transcription factor, was also reduced in rapidly progressing ALS patients [223]. In line with earlier reports, recently it was demonstrated that Tregs isolated from ALS patients’ blood were less effective in suppressing responder T cell proliferation. Although both slowly and rapidly progressing ALS patients showed Treg dysfunction, Tregs from rapidly progressing patients were affected to a greater extent [224]. Epigenetically, the methylation of the Treg-specific demethylated region (TSDR) was also greater in ALS patients compared to the healthy controls. The report further suggested functional Treg deficit as a key etiological factor in ALS. In vitro expanded ALS Tregs regained suppressive activity suggesting that passive transfer of autologous expanded Tregs might slow down disease progression. Supporting that possibility, infusion of ex vivo expanded autologous Tregs was found safe and well-tolerated in ALS patients associated with slowed disease progression rates during both early and late stages [225]. Therefore, therapies to expand Tregs can be developed for the potential treatment of ALS to suppress peripheral as well as CNS inflammatory immune responses. Recently, treatment of IL-2/IL-2-antibody complex (IL-2c) amplified endogenous Tregs in SOD93A mice. Treg expansion significantly slowed disease progression and increased survival time in mice. Treg expansion was associated with motor neuron function restoration and microglia/astrocyte inactivation with concomitant upregulation of FOXP3 and Gata3 in the spinal cord and sciatic nerve [207]. Several clinical attempts are underway to amplify Treg frequency in ALS patients. The phase II trial, known as MIROCALS, is currently ongoing to assess safety and efficacy of low-dose IL-2 as a Treg enhancer for controlling neuroinflammation in newly diagnosed ALS patients (NCT03039673). Another phase II clinical trial to determine safety and efficacy of dimethyl fumarate (Tecfidera) in patients with ALS is underway (ACTRN12618000534280). Dimethyl fumarate is currently used for the treatment of relapsing MS to increase Treg frequency and reduce inflammatory responses. Therefore, investigators are hoping to slow ALS progression by targeting the same pathway. Another candidate, fingolimod, reduces inflammation in ALS by sequestering T cells inside the lymph nodes, therefore inhibits T cell CNS entry. In a Phase II clinical trial, fingolimod was safe and sequestered lymphocytes but unfortunately sequestration also included Tregs [226]. Although, lower doses of fingolimod in mice kept Tregs in the circulation, this dose is not safe in humans due to potential heart complications [227]. Clinical studies to expand peripheral Tregs for the potential treatment of ALS are summarized in Table 2. Overall, as illustrated in Fig. 4, distinct effects of Teffs and Tregs determine ALS pathogenesis progression rate.

**Table 2:** Clinical studies to increase Treg frequency and function in different neurodegenerative disorders

Clinical Phase	Intervention	Condition	Status	Trial identifier	Outcome
I & II	Autologous Treg (GB301)	AD	Not yet recruiting	NCT03865017	–
II	Sargramostim (rhGM-CSF)	AD	Completed	NCT01409915	Improved memory function [181]
–	Dimethyl fumarate (Tecfidera)	MS	Approved	NCT02461069	Approved as first line monotherapy
–	Fingolimod (FTY720)	MS	Approved	NCT00333138	First orally approved therapy
II	Low dose IL-2	MS	Recruiting	NCT02424396	–
NA	Vitamin D3	MS	Completed	NCT00940719	Unaffected Tregs [228]
II (WIRMS)	Hookworm larvae	MS	Completed	NCT01470521	–
I	Sargramostim (rhGM-CSF)	PD	Completed	NCT01882010	Improved motor function [24]
I	Sargramostim (rhGM-CSF)	PD	Active	NCT03790670	–
I	Autologous Treg with IL-2	ALS	Completed	NCT03241784	Slow disease progression [225]
II	Autologous Treg with IL-2	ALS	Recruiting	NCT04055623	–
II (MIROCALS)	Riluzole with IL2 and 5% glucose water solution	ALS	Recruiting	NCT03039673	–
II (TEALS)	Dimethyl fumarate (Tecfidera)	ALS	Not yet recruiting	ACTRN12618000534280	–

### Stroke

Stroke is the leading cause of death and disability worldwide. Ischemic stroke is the most common type of stroke associated with cerebral ischemia and inflammatory immune responses that induce long-term secondary brain injury after initial primary brain injury [229]. Reactive astrocytes are the most characteristic feature of ischemic stroke resulting in glial scar formation at the site of tissue injury (Fig. 4). Although the exact etiology is still not clear, inflammation is considered to play a significant role in the pathogenesis of ischemic stroke that arises after apoptotic cell death of the ischemic tissue [230]. Understanding post-stroke inflammatory responses will enable researchers to understand potential therapeutic targets for stroke management and functional recovery of the affected brain. Involvement of T cell subsets in inflammation-mediated ischemic brain injury has been profoundly recognized. Following ischemic brain injury, T lymphocytes become activated, extravasate into the brain parenchyma, and accumulate in the necrotic core and ischemic penumbra [231]. Both human and animal studies showed infiltration of innate immune cells, neutrophils [232], macrophages [233], and adaptive immune T lymphocytes [234, 235] that release cytokines in response to debris from the necrotic tissue and signals from resident innate immune cells in the ischemic brain. Intracellular components from the necrotic tissue such as HMGB1, ATP, NAD and HSP70 among others serve as antigenic peptides that interact with the TLRs on infiltrating and resident innate immune cells to build a strong pro-inflammatory environment [236]. Pro-inflammatory mediators released from innate immune cells and infiltrated T lymphocytes orchestrate secondary injuries in the affected brain [237, 238]. It was shown that increased IL-17 expression colocalized with the neuroglia cells in the rat brain following permanent middle cerebral artery occlusion (MCAO) after an hour after injury. For the first time, similar IL-17 expression was observed in human ischemic brains that peaked slightly earlier suggesting the pathogenic role of IL-17 secreting pro-inflammatory cells in disease progression [239]. Later, increased infiltration of IL-23-secreting macrophages and IL-17-expressing T lymphocytes was shown in mouse brains following ischemia-reperfusion injury. To determine the functional significance of cytokines, cytokine-deficient mice that were genetically deficient (knockout) in IL-17, IL-23p19 and IFN-γ were studied. A significant reduction in infarct size was seen in IL-23p19 and IL-17 knockout mice but not in IFN-γ knockout mice. These data helped to confirm the roles of IL-23 and IL-17 in ischemic inflammatory responses [240]. Increased CNS infiltration of Teffs occurs after initial peripheral adaptive immune activation. Clinical studies have shown elevated frequency of peripherals Th17 cells in patients that corelated to the ischemic brain area [239]. A similar increase in peripheral Th17 cell differentiation with concomitant increase in pro-inflammatory cytokines IL-17A, TNF-α, IL-6 and IL-1β was observed after MCAO in mice [241]. Therefore, early peripheral Teff responses are involved in worsened later ischemic stroke outcomes. Luo et al. observed increased infiltration of both Th1 and Th17 cells in the ischemic core of the mouse brain following MCAO. Treatment with IL-33, an IL-1 family member, significantly inhibited neuroinflammation by an immune-shift of pro-inflammatory Th1 cells towards anti-inflammatory Th2 cells and suppressed Th17-mediated immune responses [241] suggesting candidates exhibiting the potential to transform effector immune responses into regulatory phenotype can be harnessed for therapeutic gain. Infiltrating Teffs can be detected for as long as 30 days post infarction in the brain parenchyma [242]. Together, the above reports suggest an indispensable role of Teffs in the ischemic neuroinflammation and Th1 and Th17 cells serve as primary invaders to the ischemic parenchyma from the periphery.

The role of immunosuppressive Tregs in the pathogenesis of stroke remains not well-defined. Few Tregs are observed early after stroke onset, but their count increases in later disease phase that last up to a month after injury. However, CD4+CD25+ Treg depletion did not affected neurological outcomes [243] and may be due to larger infarct size. In another study, selective depletion of Tregs dramatically reduced infarct size and improved neurological function 24 h after stroke onset, but this protective effect was abrogated at later disease stages [244]. Several studies, however, suggest a beneficial role of Tregs in stroke pathology. Depletion of FOXP3+ Tregs increased delayed damage and worsened functional outcomes in MCAO mice. Treg depletion also augmented post-ischemic activation of resident and infiltrated immune cells including microglia and TNF-α- and IFN-γ-secreting T cells [245] suggesting an ability of Tregs to suppress effector innate and adaptive immune responses during disease. In another study, Treg depletion completely eliminated neuroprotection afforded by IL-2c in mice [208]. A clinical study also showed a significant reduction of peripheral Teffs and Tregs in patients with acute stroke. Teff decline was linked to stroke-associated infection; whereas, Treg decline was directly associated with stroke onset [246]. Overall, neuroprotective responses offered by Tregs are notable in ischemic stroke. Indeed, adoptive transfer of Tregs during both early and late disease stages markedly reduced infarct volume and improved neurological functions in an ischemic mouse model with other long-term protective effects. Tregs restored disruption of BBB integrity in early disease stage and attenuated cerebral inflammation by affecting peripheral immune cell trafficking [202]. During early disease stage, Tregs can elicit neuroprotective responses without entering brain parenchyma. This is due to the effects on the peripheral effector immune system. By suppressing elevated IL-6 and TNF-α levels in the blood, Tregs abrogated systemic inflammatory immune responses [201]. Recently, CCR5 was found to be essential for trafficking Tregs to the injury site as CCR5−/− Tregs failed to attenuate brain infarction or neurological deficits. In contrast, CCR5-inducing Tregs inhibited BBB impairment and abrogated peripheral T lymphocyte trafficking [122]. Therefore, therapeutic approaches to expand peripheral Tregs could be advantageous to suppress both peripheral and central immune activation in stroke. Boosting Treg immunosuppressive capacity and IL-10 expression using HDACi reduced infarct volume, behavioral deficits, and cerebral neuroinflammation in a cortical ischemia mouse model [27]. Beneficial effect of IL-2c on Treg expansion was shown in a rodent model of ischemic stroke. In the periphery, IL-2c elevated Treg numbers in blood, spleen, and lymph nodes and boosted their Teff suppressive function by enhancing expression of CD39 and CD73. In the brain, elevated Tregs reduced infarct volume and neuroinflammation and improved sensorimotor functions [208]. Figure 4 illustrates the different roles of Teffs and Tregs in the pathogenesis of stroke by emphasizing the neuroprotective Treg role.

### HIV-1 associated neurocognitive disorders (HAND)

With antiretroviral therapy (ART), the life expectancy of HIV-1 infected people has increased significantly. However, end organ disease has continued almost unabated with low level infection inciting, for example, HIV-1 associated neurocognitive disorders (HAND). Up to 50% of ART-treated HIV-1 infected people develop HAND [196]. The disease spectrum includes asymptomatic neurocognitive impairment, mild neurocognitive disorder, and HIV-associated dementia [197]. Increasing evidence demonstrates that a low CD4+/CD8+ ratio is associated with an increased risk of disease in virally suppressed infected individuals [199, 200]. Indeed, reduced CD4+/CD8+ ratio in both peripheral blood and CSF correlate with the neurocognitive impairment scores of ART-treated HIV patients. These data support the idea that HIV patients with low CD4+ T cell counts are at greater risk of developing HAND compared to patients with higher cell counts. Additional analysis of T cell subsets revealed that reduced frequency of naïve CD4+ and CD8+ T cells were associated with increased frequency of effector memory CD4+ and CD8+ T cells in HIV patients compared to healthy controls [203]. Reduced Treg frequency has been reported in HIV-infected individuals with low CD4+ T cell counts [204]. Others demonstrated increased frequency of Tregs with reduced HIV-specific immunosuppressive functions in patients with low CD4+ T cells on ART compared to patients left untreated [205]. High levels of circulating Th17 cells were observed in HIV patients with poor CD4+ reconstitution despite being on ART treatment. Interestingly, this occurred with increased Treg frequency and immune activation [206]. Other studies reported no differences in expression of both CD4+CD25+ and FOXP3+ Tregs in HIV patients before and after ART. However, HIV patients had higher Treg percentages compared to healthy controls [209]. Despite such discrepancies, most studies reported increased Treg frequency among different CD4+ T cell subsets during HIV infection irrespective of ART [210–212].

The consequences of increased Treg numbers during HIV could be either beneficial by abrogating generalized T cell activation preserving CD4+ T cell counts or detrimental by weakening antiviral immune responses and affecting viral persistence [212]. Previously, neuromodulatory effects of Tregs were demonstrated. Intracranial injection of human HIV-infected murine bone marrow-derived macrophages induced focal encephalitis with robust micro- and astrogliosis in mice. Adoptive transfer of Tregs significantly attenuated neuroinflammation and led to neuroprotective responses [228]. A subsequent study demonstrated that Tregs migrated to the virus-induced neuroinflammatory sites and modulated microglial responses in HIV-1 encephalitis [247]. Tregs can kill HIV-1 infected macrophages as well as transform them from a neurodestructive to a neuroprotective phenotype [248]. Tregs suppress HIV-1 infection in CD4+ T cells by cell contact [249]. Suppression of HIV-specific responses occur throughout the course of HIV infection are attributed to lymphoid Tregs [250]. Notably, Tregs present in newborns from HIV-infected mothers can escape infection. This suggests a potential role of Treg immunomodulation in mother-to-child HIV transmission [251]. These reports support the beneficial role of Tregs in infection by suppressing HIV-specific immune responses. In contrast, several studies suggest a detrimental role of Tregs in HIV. In one, IL-2 therapy along with ART was used to expand CD4+ T cells in patients beyond what was seen by ART alone. However, despite sustained CD4+ cell numbers, improved clinical outcomes were not seen [252]. The results were attributed to IL-2-mediated Treg induction [253]. Studies suggest that circulating Tregs are now readily susceptible to HIV infection [210, 254]. Immune activation and viral rebound following ART interruption lead to Treg expansion [255]. Studies have shown higher frequency of inducible, intact proviruses in Tregs compared to other CD4+ T cell subsets suggesting Tregs as a key latent HIV reservoir [256–258]. Thus, the role of Tregs in HIV pathogenesis may depend on disease stage.

Overall, affecting peripheral Tregs is a new frontier in neurodegenerative disease treatment. It can control systemic as well as CNS inflammatory processes. A list of candidates capable of expanding peripheral Tregs that have been tested are summarized in Table 1. Clinical trials targeting peripheral Tregs are also summarized and appear in Table 2.

## Conclusions

The brain is no longer considered an immune-privileged organ. Cross talk between peripheral and CNS immunity sustains homeostasis. While few T cells traffic into the CNS they have profound effects on brain function [5]. During infectious, metabolic, degenerative and immune-mediated brain injury, the homeostasis of the brain-immune axis is disturbed with the emergence of effector immune populations and expanded inflammatory activities [11]. Over the past decades, the essential contributions, in health or disease, of the adaptive immune system in brain function has been well-established [2, 13, 14]. The driver affecting the balance between pro-inflammatory Teffs and anti-inflammatory Tregs is the disease itself. Therefore, peripheral emergence of effector immune cells (for disease) and downregulation of regulatory immune cells (protection) may represent an early disease biomarker [3]. To reflect human-specific immune responses operative in brain disorders, our laboratory created a human IL-34 (hIL-34) expressing transgenic NOG mice (NOD.Cg-*Prkdc*^*scidI*^*l2rg*^*tm1Sug*^Tg(CMV-IL34)1/Jic). Upon human hematopoietic stem cell reconstitution, mice develop a human immune system along with human “microglia-like” cells in the brain [259]. Introduction of human AD transgenes or neurotoxins in hIL-34-NOG mice serve to explore human disease-specific immune alterations after exposure to misfolded and aggregated proteins and how immunity can be harnessed to modify disease outcomes. Therapeutic strategies to boost expansions of immunoregulatory, anti-inflammatory, and neuroprotective Tregs are a promising venue to affect neurodegenerative disease progression by sustaining neuroprotective immunity.

## Data Availability

Not applicable.

## Abbreviations

Aβ: Amyloid-beta
AD: Alzheimer’s disease
ALS: Amyotrophic lateral sclerosis
APCs: Antigen presenting cells
APP: Amyloid precursor protein
AQP4: Aquaporin 4
ART: Antiretroviral therapy
BBB: Blood-brain barrier
BDNF: Brain-derived neurotrophic factor
bvPLA2: Bee venom phospholipase A2
CAM: Cell adhesion molecules
CNS: Central nervous system
CSF: Cerebral spinal fluid
CTLs: Cytotoxic T lymphocytes
CTLA4: Cytotoxic T lymphocyte antigen 4
DC: Dendritic cell
EAE: Experimental autoimmune encephalomyelitis
FOXP3: Forkhead box protein P3
GM-CSF: Granulocyte-macrophage colony stimulating factor (GM-CSF)
GDNF: Glial cell-derived neurotrophic factor
HAND: HIV-1 associated neurocognitive disorders
HDACi: Histone deacetylase inhibitor
hUC-MSCs: Human umbilical cord-derived mesenchymal stem cells
ICAM-1: Intercellular adhesion molecule-1
IFN-γ: Interferon gamma
IGF-1: Insulin-like growth factor 1
IL-2: Interleukin 2
IVIg: Intravenous immunoglobulin
LFA: Leukocyte function-associated antigen
MBP: Myelin basic protein
MCAO: Middle cerebral artery occlusion
MCI: Mild cognitive impairments
MHC: Major histocompatibility complex
MMSE: Mini-mental state examination
MPTP: 1-methyl-4-phenyl-1,2,3,6-tetrahydropyridine
N-α-syn: Nitrated alpha synuclein
MS: Multiple sclerosis
NFTs: Neurofibrillary tangles
NK: Natural killer
NLR: Nucleotide-binding oligomerization domain-like receptors
PD: Parkinson’s diseases
PLP: Myelin proteolipid protein
PRRs: Pattern recognition receptors
PS1: Presenilin 1
ROS: Reactive oxygen species
SCFA: Short-chain fatty acids
SIRPA: Signal regulatory protein α
SN: Substantia nigra
SNpc: Substantia nigra pars compacta
SOD1: Superoxide dismutase 1
TCR: T cell receptor
TGF-β: Transforming growth factor beta
Teffs: Effector T cells
Th: T helper
TLR: Toll-like receptor
TNF: Tumor necrosis factor
Tregs: Regulatory T cells
Tres: Responder T cells
TSDR: Treg-specific demethylated region
UPDRS-III: Unified Parkinson’s Disease Rating Scale-III
VIP: Vasoactive intestinal peptide
